# Amplicon Sequencing Minimal Information (ASqMI): Quality and Reporting Guidelines for Actionable Calls in Biodefense Applications

**DOI:** 10.1093/jaoacint/qsad047

**Published:** 2023-04-17

**Authors:** Ishi Keenum, Robert Player, Jason Kralj, Stephanie Servetas, Michael D Sussman, Joseph A Russell, Jennifer Stone, Sailaja Chandrapati, Shanmuga Sozhamannan

**Affiliations:** National Institute of Standards and Technology, Biosystems and Biomaterials Division, Complex Microbial Systems Group, Gaithersburg, MD 20899, USA; The Johns Hopkins University, Applied Physics Laboratory, Laurel, MD 20723, USA; Datirium, LLC, Cincinnati, OH 45526, USA; National Institute of Standards and Technology, Biosystems and Biomaterials Division, Complex Microbial Systems Group, Gaithersburg, MD 20899, USA; National Institute of Standards and Technology, Biosystems and Biomaterials Division, Complex Microbial Systems Group, Gaithersburg, MD 20899, USA; US Department of Agriculture, Agricultural Analytics Division, Livestock and Poultry Programs, Agricultural Marketing Service, Washington, DC 20250 USA; MRIGlobal, Gaithersburg, MD 20878, USA; MRIGlobal, Gaithersburg, MD 20878, USA; Neogen Food Safety, Maplewood, MN 55125, USA; Joint Program Executive Office for Chemical, Biological, Radiological and Nuclear Defense (JPEO-CBRND), Joint Project Lead for CBRND Enabling Biotechnologies (JPL CBRND EB), Frederick, MD 21702, USA; Joint Research and Development, Inc., Stafford, VA 22556, USA

## Abstract

**Background:**

Accurate, high-confidence data is critical for assessing potential biothreat incidents. In a biothreat event, false-negative and -positive results have serious consequences. Worst case scenarios can result in unnecessary shutdowns or fatalities at an exorbitant monetary and psychological cost, respectively. Quantitative PCR assays for agents of interest have been successfully used for routine biosurveillance. Recently, there has been increased impetus for adoption of amplicon sequencing (AS) for biosurveillance because it enables discrimination of true positives from near-neighbor false positives, as well as broad, simultaneous detection of many targets in many pathogens in a high-throughput scheme. However, the high sensitivity of AS can lead to false positives. Appropriate controls and workflow reporting can help address these challenges.

**Objectives:**

Data reporting standards are critical to data trustworthiness. The standards presented herein aim to provide a framework for method quality assessment in biodetection.

**Methods:**

We present a set of standards, Amplicon Sequencing Minimal Information (ASqMI), developed under the auspices of the AOAC INTERNATIONAL Stakeholder Program on Agent Detection Assays for making actionable calls in biosurveillance applications. In addition to the first minimum information guidelines for AS, we provide a controls checklist and scoring scheme to assure AS run quality and assess potential sample contamination.

**Results:**

Adoption of the ASqMI guidelines will improve data quality, help track workflow performance, and ultimately provide decision makers confidence to trust the results of this new and powerful technology.

**Conclusion:**

AS workflows can provide robust, confident calls for biodetection; however, due diligence in reporting and controls are needed. The ASqMI guideline is the first AS minimum reporting guidance document that also provides the means for end users to evaluate their workflows to improve confidence.

**Highlights:**

Standardized reporting guidance for actionable calls is critical to ensuring trustworthy data.

Amplicon sequencing (AS) is widely applied for microbial pathogen detection and identification (1, 2), and has emerged as a leading technique for biosurveillance applications requiring high sensitivity and specificity (3). AS is preferred over traditional PCR approaches for its method performance, e.g., lower LOD compared to PCR alone. Sequencing PCR products (amplicons) can rapidly confirm the PCR target without size separation reducing the risk of false negatives and positives (see Supplemental Text S1). AS can also enable higher throughput than quantitative PCR (qPCR) which is limited to only four fluorophores (probes) per sample in order to achieve a distinct wavelength band signal per unique amplification target. Reliable detection has been reported in a limited multiplex format qPCR assay (2500 to 50 000 CFU/mL) for a variety of bacterial targets, compared to 10 to 50 CFU/mL for AS (4). When the goal is qualitative detection of a particular pathogen, e.g., an outbreak investigation, AS with pathogen-specific primers is an incredibly powerful tool and can provide genomic enrichment in low-abundance samples, while the sequencing allows for variant identification (single nucleotide variants, indels, etc.) within the amplified region. Amplified sequence targets can then go on to be used for characterizing genetic drift in the target pathogen (5), enabling phylogenetic studies (1), near-neighbor discrimination (6), and leveraged for primer optimization. While the U.S. Food and Drug Administration (FDA) provides some guidelines on AS-based diagnostics (7), there are currently no specified standards or guidelines for the implementation of AS to support actionable calls in biosurveillance. Consistent with current literature including diagnostic guides, we identify specific requirements applicable to biosurveillance/biodetection of microbial pathogens.

Specific guidelines and minimum requirements, including appropriate controls, are critical for data quality, and underpin confidence in analytical workflows to enable actionable results. AS methods require multi-step workflows consisting of sample collection and handling, nucleic acid extraction, PCR amplification, library preparation, and sequencing and bioinformatic processing (Figure 1). Due to the sequential nature of the workflow, systematic errors can be compounded. Minimum information guidelines for reporting and QA/QC exist for qPCR (MIQE; 8), genome sequences (MIGS; 9), marker gene sequences (MIMARKS; 10), and environmental monitoring (Environmental Microbiology Minimum Information [EMMI]; 11); however, these guidelines do not extend to AS approaches. Past work by Murray et al. (12) also identified the need for further development of AS workflow guidelines as bench methods and controls are often ignored. PCR is the current gold standard for detection of biodefense pathogens, and the further implementation of AS helps address several limitations including: (1) false-negative results due to target sequence variations, (2) false-positive results due to near-neighbor target matches, and (3) variable amplification efficiencies impacting LOD measurements. However, both methods are still impacted by inhibitors.

**Figure 1.**
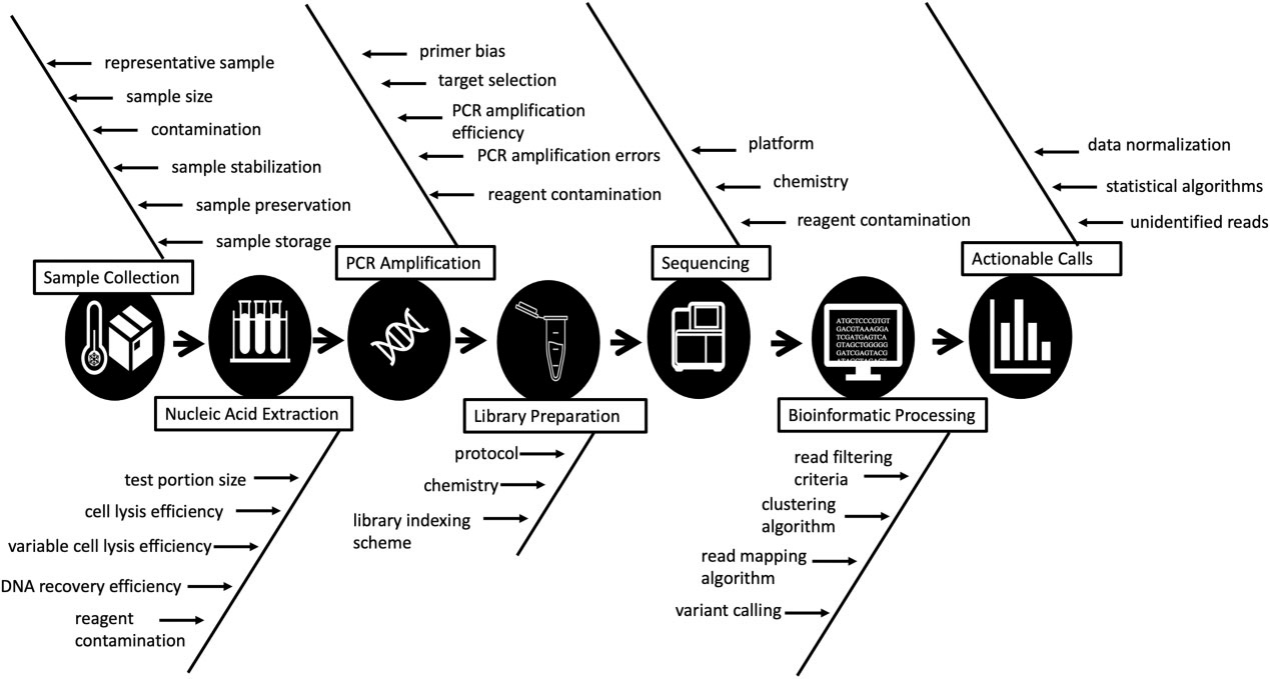
Known workflow steps that introduce biases in an AS workflow.

The objective of this document is to outline a set of requirements for AS workflows. The Amplicon Sequencing Minimal Information (ASqMI) details reporting guidelines and controls that will support reproducibility in day-to-day operations by providing guidance to mitigate/capture biases and enable actionable calls based on robust sequencing data from operational samples. AOAC INTERNATIONAL has a long-standing tradition in developing *Standard Method Performance Requirements* (SMPRs^®^) and has developed many SMPRs for agent detection assays and methods in the past; unfortunately, there are no guidelines for data elements, collection, and reporting for AS assays. Hence, we used the AOAC model to generate these guidelines and an SMPR will be developed in the future based on the ASqMI guidelines. Herein, we have provided platform-agnostic guidelines as much as possible, allowing and requiring the end user to optimally adapt these guidelines to their specific operational requirements. Additionally, we have provided routine and process checklists for laboratories to use to identify key elements of QA/QC in an AS workflow (Supplemental Worksheet 1). The objective of the Process Reporting Checklist is to provide end users with a comprehensive list of metadata that should be recorded to enable better troubleshooting when methods and controls do not perform as expected. A mock data set is provided along with an example reporting sheet, to serve as a guide (Supplemental Worksheet 2). Laboratories can utilize the point system to begin to gain consensus on their routine performance and identify potential areas for improvement.

## Challenges with Sample Matrices

The EMMI guidelines are a good source for sample collection controls. Although they will not be covered in detail here (11), we would like to emphasize the impact of sample matrix on the interpretability of subsequent results and conclusions. Broadly, samples for biosurveillance can be separated into two categories: complex background, high bioburden (e.g., soils, wastewater) and low genomic yield samples (e.g., aerosol collection filters). Sample processing and bioinformatic workflows vary depending on the expected complexity of the sample (thresholds and the stringency for near-neighbors may need adjustment), as well as the amount of recoverable DNA or RNA. Ultimately, the sample matrix will greatly influence the conclusions, and it is critical that matrix effects are considered during workflow development. It is critical to well document the sampling method so that if field blanks are positive (Supplemental Worksheet 1), it is possible to identify how contamination could have occurred in the field.

### The Need for Controls

**Figure 2.**
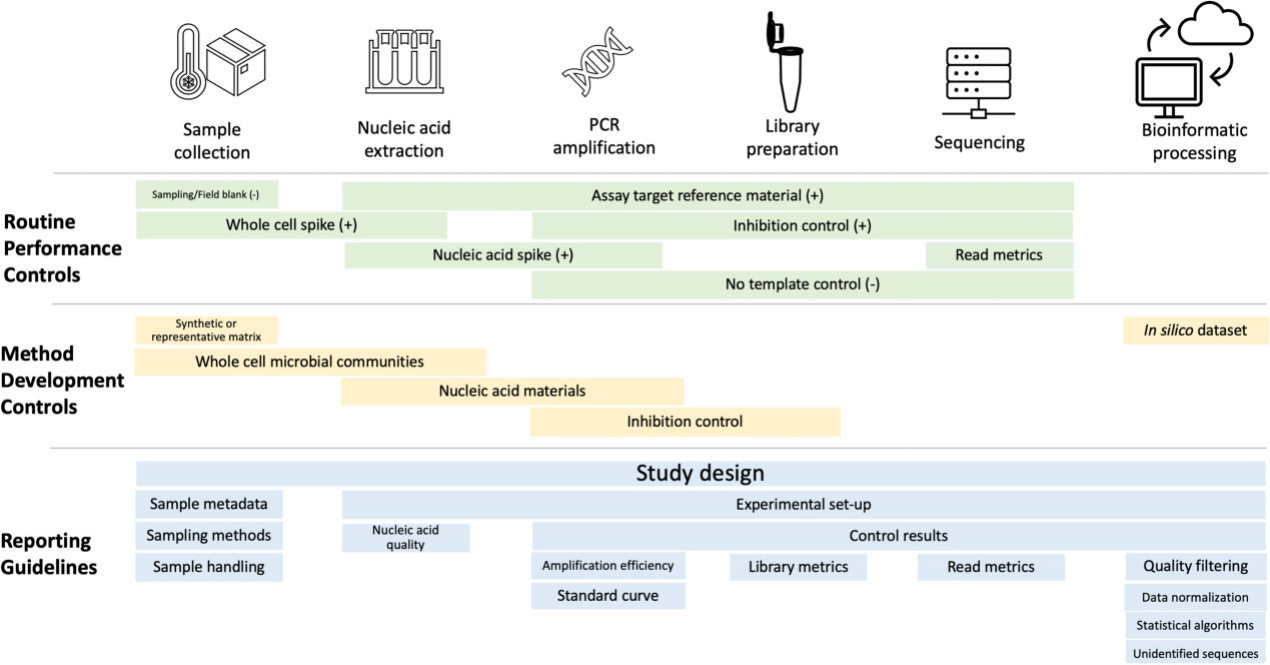
Summary of controls and reporting guidelines for an AS workflow.

Every step in an AS workflow, from sampling to data processing, can introduce systematic errors (13–16; Figure 1). Method and material controls are important for allowing the characterization of these systematic errors (Figure 2). Controls should be implemented at multiple stages in the sampling-to-analysis workflow to enable the identification of potential contamination (see Supplemental Text S2). Commonly, one control introduced early in the workflow can serve as a verification for downstream steps.

### Negative Controls

Negative controls such as sampling blanks (matrix blanks taken to the field and opened with each sample collected to account for sampling-based contamination) and no template controls (NTCs), serving as true-negative controls, should always be included in AS methods to control for false positives and ensure confidence. As a minimum, three biological sample replicates should be included to enable the practical calculation of the mean and SD of aligned read counts. If any NTC sample contains reads that align to a target reference above a practical threshold for a positive result, then these metrics indicate the aligned read counts for an amplicon are the result of contamination or another issue (17). For example, a threshold used in the Diagnostic targETEd seQuencing adjudicaTion analysis software uses three SDs (95% confidence) above the mean aligned read count of the NTCs for an amplicon reference as the minimum threshold that a sample needs to exceed for that same amplicon to be reported as detected in a sample (18).

### PCR Assay Controls

These guidelines are focused on detection and identification of specific targets discriminating between a narrow range of microbes, potentially including a subset of a species or strain, in contrast to more general, broad target AS schemes such as 16S rRNA. AS method validation controls should always include ground truth material containing true positives (or simulated true positives) and true negatives to ensure PCR primer performance. Primer performance should be validated in the same format that will be used in the method, e.g., a multiplex assay. Initial primer performance validation serves as a go/no-go screen for the primer assays and should be evaluated to confirm the correct band size and specificity. Synthetic positive controls with unique barcodes for target amplicons that are distinguishable by sequence content can be used in the PCR workflow. It is critical to further interrogate sample positive or negative controls that are not performing as intended to identify where the workflow can be better optimized or where laboratory contamination is being introduced (Supplemental Worksheet 1).

### Inhibition Control

Assay performance should also be evaluated in the specific matrix of interest to assess the contribution of inhibitors. When isolating nucleic acids from complex environmental matrices such as soil or wastewater, it is critical to consider whether there are inhibitors present in the sample that could disrupt the performance of PCR amplification leading to false negatives (19). There are multiple ways to detect the presence of inhibitors including internal amplification controls, parallel amplification controls, and dilution curves. Inhibition controls should be included in the method for all sample types and from each sample site because differing soils and environmental locations will have different levels and types of inhibitors. A dilution curve can be used to select an optimal sample dilution depending on balancing the inhibitors detected and the expected variability and abundance of target gene copies (i.e., that which maximizes signal). Supplements, such as proteinase inhibitors, can also be added to the qPCR reaction to enhance amplification (20).

## Library Preparation and Amplicon Sequencing

Library preparation (LP) compatibility with the sequencing platform is important. In general, best practices that apply to all AS LP methods include: (1) minimizing amplification cycles to obtain sufficient yield without introducing unnecessary amplification errors and bias, (2) reducing cross-contamination risk by minimizing LP steps and transfers, and (3) using high-fidelity polymerases compatible with the LP chemistry (21, 22). (*Note:* If deoxy-UTP [dUTP] is included in initial PCR amplification, any subsequent PCR must be performed with a dUTP-compatible polymerase.)

### Library Preparation

Most AS LP methods and sequencing platforms require DNA purification steps to remove extraneous oligonucleotides (e.g., primers, adaptors), as well as other reaction components (e.g., proteins, salts) that can interfere with downstream processes. LP batches should include appropriate control samples/libraries to monitor for cross-contamination, because there is a potential risk for contamination at this step (23). The yield and fragment size of the LP should be assessed not only to ensure that the LP was successful, but to determine the volume of library material that should be loaded onto the sequencer and allow for sample concentration normalization when multiple indexed/barcoded libraries will be sequenced together. Although some LP methods make yield and size determination optional, e.g., by using bead binding methods to normalize and limit loading concentrations, a pre-sequencing analysis of libraries to ensure adequate yield and representation on the sequencing run is a critical QC measure, especially in the field of biodefense, where high-confidence calls are a requirement. Consistently evaluating the performance of the LP method is critical for confidence in the AS results (Supplemental Worksheet 1).

### Sequencing

Laboratories performing PCR and next-generation nucleotide sequencing methods may already have the existing required equipment or are planning to purchase new equipment. A number of next generation sequencing platforms are suitable for AS. Factors to consider when selecting a platform include: throughput, cost, turnaround time, ease of use, contamination risks, laboratory setting (mobile or stationary), amplicon length, and assay type (low-level or no multiplexing versus highly multiplexed, and presence/absence versus sequencing of an informative region).

Additionally, sequencing objectives dictate the required number of reads and read length. This could mean adjusting the number of cycles for synthesis-based sequencers or adjusting the run time for direct detection sequencers. More reads may be necessary to obtain high-confidence calls when read quality is low, when the assay is highly multiplexed (multiple amplicons competing for coverage), and for mixed/complex samples that may produce different amplicon sequences for the same region/assay. Regardless of sequencer and assay specifics, quality checks should be performed that include confirmation that sequence quality scores meet expectations for the sequencer, and that the number of reads produced per run—and more importantly per sample—are adequate to meet analytical objectives.

Depending on the sequencing approach, users should monitor and mitigate the possibility of on-instrument “contamination.” This includes intra-run crossover via read mis-assignment (e.g., index hopping; 24), as well as inter-run carryover for some sequencers that wash and re-use fluidics, flow-cells, or other components exposed to sample libraries. Although typically occurring at very low frequencies, even a very small percentage of crossover/carryover from a sample with a highly abundant amplicon can be significant, once again highlighting the need for contamination monitoring using control samples, and the importance of considering both laboratory context and appropriate detection thresholds during data analysis.

## Bioinformatic Processing

### Assessing Read and Base Call Quality

Sequence quality is a critical factor to ensure accurate detection with AS methods, and one of the most important factors missing from many repositories (25, 26). AS using any system can produce high quality results (27, 28). Accurate single-nucleotide base calls enable the identification of rare single-nucleotide polymorphisms, often important in near-neighbor discrimination (29). Base calling algorithms differ by the type of sequencing used. Understanding the different algorithms used for base calling can help identify relevant read quality thresholds. Quality scores indicate the amount of measurement uncertainty present for a base call (30, 31). For example, for Phred (Q) scores, high-quality scores indicate a low probability of error. Low-quality scores indicate a significant portion of the read is unusable. If used, it may lead to false variant calls and inaccurate conclusions. A related AOAC Stakeholder Program on Agent Detection Assays (SPADA) effort, lays the framework for read quality and reference sequences used in biothreat agent detection, identification, and quantification.

### Sequence Cleanup and Filtering

Quality control of AS read data begins with removing adapters and contaminating sequences from the raw reads. A set of read metrics (e.g., reads generated, quality scores [Supplemental Worksheet 1]) should be reported and acceptable criteria for each metric should be defined before beginning the analysis for both reference (32) and amplicon target sequences.

### Read Classification and Database Selection

Classification is the process of assigning a given quality-filtered sequence from an amplicon to the most closely matching representative sequence in a database of reference genomes, genome segments, or amplicons. The AS classification problem presents unique challenges, including: (1) a sequencing platform error rate that can be mis-assigned as meaningful biological variation in short target sequences; (2) pair-wise sequence alignments that can constrain or obscure sequence-based information; and (3) ensuring appropriate resolution amongst clades sharing high genome homology. Choosing the most appropriate classification tool for a given application can help ameliorate some of these challenges. There are a myriad of alignment and classification tools available, and each tool has strengths and weaknesses. For these reasons, and given the rapid development in this space, the authors cannot prescribe a specific tool. Rather, we offer some suggestions for selecting the appropriate tool (see Supplemental Text S3). Options include traditional sequence-composition (e.g., k-mer matching [33–36]) and alignment-based tools (37–42). Additionally, newly emerging machine learning methods show increasing promise (43, 44).

The database requirements for AS approaches are somewhat straightforward as the goal is to map the reads from a known selected target following PCR amplification. The quality of the database alignment can be established based on preset criteria, and these presets should be based on the known similarities of near-neighbor sequences in the non-targeted organisms (e.g., perfect [100% match over the entire length of the amplicon] or conditional [≥90% match over ≥90% of the amplicon sequence]). In a complex sample, identifying targeted versus near-neighbor organisms solely based on AS may pose some challenges; therefore, the reference database should contain whole-genome sequences of the targeted organisms as well as whole-genome sequences of organisms that can be found in the sample matrix enabling read mapping to an inclusivity and exclusivity set. The latter may aid in identifying non-target reads (reads that potentially align to other parts of the genome as well as those that can align to multiple genomes from the target environments). When including whole-genome sequences it is recommended to follow the sequence quality recommendations established by the ISO standard 23418 and AOAC SPADA SMPR (32).

## Creating Response Actionable Calls

### Establishing Thresholds for Action

Amplicon-based detection is suited to qualitative detection (i.e., returning a binary presence/absence result), rather than quantitative detection unless system response is characterized using a controlled dosage of the target. Prescribing read counts for detection cutoffs is not currently feasible due to the number of workflow variables.

Appropriate detection thresholds rely on both (1) pathogen levels associated with an increased risk and (2) the LOD or probability of detection of analyte in a defined matrix (45, 46) (see Supplemental Text S4). The threshold for risk depends on the pathogen, and ranges from any level above the LOD to some prescribed level (e.g., fecal coliform monitoring in recreation waters). Users must also consider how to treat ambiguous results based upon an organism’s risk and historical data (see Supplemental Text S5). If control experiments produce starkly different signals (e.g., read counts) for the positive and negative controls, but a test sample produces a signal between those, then one must have a plan for interpreting the results.

Finally, time requirements are another variable/limitation that must be considered. Real-time monitoring and analyses of sequence read data is feasible in Oxford Nanopore Technology (ONT) sequencing which does not have a fixed time for analysis. For routine monitoring, establishing a fixed run time and/or number of reads is good practice to ensure consistent monitoring and reduce the likelihood of false positives. One may also establish the practice where detection of some pre-established number of “hits” before a given time window would also constitute a positive signal. Because amplicon workflows pre-amplify targets, one could establish an LOD based upon threshold run time using well-controlled numbers of target copies. Ultimately, a clear separation between positive and negative controls needs to be identified and the time for sequencing in a real-time workflow will be dictated by the program requirements and the concept of operations.

## Conclusions

AS workflows can provide robust, confident calls for biodetection; however, due diligence in reporting and controls are needed. The ASqMI guidelines represent the first AS minimum reporting guidance document that also provides detailed checklists of methods for end users to evaluate their workflows to improve confidence. Laboratories should employ the FAIR reporting guidelines (findability, accessibility, interoperability, and reusability) as well as ensuring that metadata is appropriately collected (47). While publicly available reporting may not be possible in many biodefense scenarios, workflows must still be validated, and this data structure should still be employed for method consistency and confidence.

## CRediT Author Statement

**Ishi Keenum:** Conceptualization; Writing—original draft, review & editing. **Robert Player:** Conceptualization; Writing—original draft, review & editing. **Jason Kralj:** Conceptualization; Writing—original draft, review & editing. **Stephanie Servetas:** Conceptualization; Writing—original draft, review & editing. **Michael D. Sussman:** Conceptualization; Writing—original draft, review & editing. **Joe Russell:** Conceptualization; Writing—original draft, review & editing. **Jennifer Stone:** Conceptualization; Writing—original draft, review & editing. **Sailaja Chandrapati:** Conceptualization; Writing—original draft, review & editing. **Shanmuga Sozhamannan:** Conceptualization; Writing—original draft, review & editing.

## Supplementary Material

qsad047_Supplementary_Data
