# Supplementary material for: Amplicon Sequencing Minimal Information (ASqMI): Quality and Reporting Guidelines for Actionable Calls in Biodefense Applications
Source: J AOAC Int. 2023 Apr 17;106(5):1424–30. doi: 10.1093/jaoacint/qsad047 (PMC10472743; doi:10.1093/jaoacint/qsad047)
Supplement: qsad047_Supplementary_Data [file qsad047_supplementary_data.zip › qsad047-suppl_data/aoac-23-0045-File004.docx]

**Supplemental Information for**

**A**mplicon **S**e**q**uencing **M**inimal **I**nformation (AsqMI): Quality and Reporting Guidelines for Actionable Calls in Biodefense Applications

Authors and Affiliations

Ishi Keenum^1*^, Robert Player^2,3^, Jason Kralj^1^, Stephanie Servetas^1^, Michael D. Sussman^4^, Joe Russell^5^, Jennifer Stone^5^, Sailaja Chandrapati^6^, Shanmuga Sozhamannan^7,8^

^1^Complex Microbial Systems Group, Biosystems and Biomaterials Division, National Institute of Standards and Technology, Gaithersburg, MD, 20899, USA

^2^ Applied Physics Laboratory, The Johns Hopkins University Laurel, MD, 20723,USA.

^3^ Datirium, LLC, Cincinnati, OH, 45526, USA
^4^Agricultural Analytics Division, Livestock and Poultry Programs, Agricultural Marketing Service, US Department of Agriculture, Washington, DC, 20250, USA
^5^MRIGlobal, Gaithersburg, MD, 20878 USA

^6^Neogen Food Safety, Maplewood, Minnesota, 55125, USA

^7^Joint Program Executive Office for Chemical, Biological, Radiological and Nuclear Defense

(JPEO-CBRND), Joint Project Lead for CBRND Enabling Biotechnologies (JPL CBRND EB), 21702, Frederick, MD, USA

^8^Joint Research and Development, Inc., Stafford, Virginia, USA

**Text S1: Comparison of PCR to Amplicon sequencing analyses**

PCR is the current gold standard for detection of biodefense pathogens; however, it can (i) be inhibited by contaminants, (ii) give false negative and false positive results due to target sequence variations and near-neighbor target matches, respectively, and (iii) varying degrees of amplification efficiencies can impact limit of detection (LOD) of the PCR method. Sequencing PCR products (amplicons) can rapidly confirm the PCR target without size separation reducing the risk of false negatives and positives. For example, qPCR will generate fluorescence data for each amplification cycle, but if a predetermined fluorescence intensity is not achieved before a certain number of cycles (often referred to as Ct values), no detection will be reported. In AS, amplicons are generated within the established cycle time and post amplification sequencing can be leveraged to not only determine the presence of a particular organism in a sample, but also identify variants (SNVs, indels, etc.) within the amplified region or even the primer sequences themselves. That is, sequencing the generated amplicons provides a richer data set than PCR alone.

AS can also enable higher throughput than qPCR which is limited to only four fluorophores (probes) per sample in order to achieve a distinct wavelength band signal per unique amplification target. Generally, this limits the number of organisms that can be tested to one or two per PCR sample, as it is often necessary to use multiple targets per organism to gain strain-level specificity. Further complexity is added because each probe sequence associated with a fluorophore can potentially cross react with other primers in the multiplex, as well as background organism genomes with complementary sequences reducing fluorescence intensity and the likelihood of a valid result. Notably, amplicon sequencing does not require fluorescent probe sequences in the amplification reaction master mix for detection, reducing the complexity of the PCR multiplex function.

A 16-plex PCR coupled with Next Generation Sequencing (NGS) using the Illumina MiSeq platform has been used for the detection of waterborne pathogens (1). Success has also been reported in another independent study with a 14-plex PCR coupled with third generation sequencing (TGS) using an Oxford Nanopore Technologies (ONT) MinION device (2).

**Text S2: Types of Controls**

Method controls are important and need to be considered for AS. The combination of positive and negative controls provide confidence that a sample identified as positive is not a false positive and that the workflow is sufficiently specific enough that environmental bacteria are not mis-identified. Here are some examples of control groups:

1. Internal controls are known entities that are added into a sample in order to confirm the efficacy of a process or workflow. These can take the form of whole cell/viral spike or nucleic acid spike.
2. Spikes are added to a sample and assessed after a processing step for their recovery. They can be useful when determining the LOD or LOQ in complex samples or for a novel workflow.
3. Parallel process controls are performed as separate samples that serve to verify either a lack of contamination or the ability to detect a target. Both internal and parallel process controls can serve as negative or positive confirmation depending on the use.
4. Negative controls allow the identification of “background noise” or the natural variation present in negative samples and can enable setting cutoff values for positive confirmation of targets. Negative controls serve to identify potential contamination and/or false positives in a workflow. Each step in a workflow should have a corollary negative control to serve to identify contamination at that stage of the workflow.
5. Positive controls serve to show that if a target is present, it can be identified with a specific workflow. A true target positive sample is important during workflow development and verification. However, processing unknown test samples and true positives together introduces unnecessary risk for false positives due to sample-to-sample (control-to-unknown) contamination. For established workflows, it is therefore critical that the positive control material can be clearly distinguishable from a “true positive test sample”. Surrogate organisms, genetically labeled sequences, and synthetic positive controls (engineered fragments that are able to be amplified by primers but contain unique intermediary sequences) that can be easily distinguished from a true target based on sequencing should be implemented to confirm the efficacy of an established workflow.

**Text S3: Alignment and Classification methods**

There are a number of alignment and classification tools available, and each of these tools has its set of strengths and weaknesses. Furthermore, there is rapid development in these bioinformatics applications. In selecting the appropriate tool, the following criteria should be considered: 1) traditional sequence-composition (e.g., k-mer matching) and 2) alignment-based tools and machine-learning (ML) methods.

Alignments with tools such as BLAST(3),bowtie (4), BWA(5) , and minimap2 (6) allow direct ‘mapping’ of reads to references. This supports direct inspection of sequence read classification based on nucleotide-to-nucleotide read identity across the length of the read, which eliminates some of the ‘black box’ paradigm associated with less interpretable ML approaches. Benefits to an alignment approach also include more flexibility with respect to reference databases, as references can be added ‘on the fly’ without the computationally-burdensome re-indexing of k-mer methods or re-training of models with ML methods. However, balancing alignment settings (e.g., match scores, mismatch penalties, gap-open penalties, etc.) for optimal sensitivity and specificity is challenging for amplicon panels that vary in the number of nucleotide differences that define a target. Allowing limited flexibility in the mapping settings to accommodate sequencing error and potential mutations in desired target regions can quickly open the door for reads to ‘multi-map’ across references, undermining specificity. Lastly, direct alignment of reads to references is much less computationally-efficient than k-mer indexing or ML methods. This can significantly inflate analytical run-times over millions of sequencing reads which may be a consideration in time-sensitive biosurveillance applications. Centrifuge (7) effectively addresses the analysis speed consideration by leveraging a hybridized Burrows-Wheeler transform and Ferragina-Manzini indexing and compression of the reference database; however, some analyses have identified poor specificity resulting from this approach in shotgun metagenomics workflows.

Classifiers based off sequence composition – e.g., k-mer hashing/matchers like the Kraken (8) and CLARK (9) families of tools – have a primary benefit of speed, allowing larger database comparisons to be tractable on operational biosurveillance timescales. Kraken2 (10) offers logistical benefits over the original algorithm by instituting a probabilistic compact hash table of ‘minimizer’ l-mers (rather than full k-mers) which lowers memory requirements and increases speed. CLARK- I (light) aims for similar logistical benefits with a much-reduced database footprint. More recent algorithms have adjusted similar approaches to be more optimal for the long and/or error-prone sequence reads from third-generation sequencing platforms (e.g., PacBio, Oxford Nanopore Technologies). MetaMaps (11) is built upon the framework of MashMap (12), which assigns reads via a k-mer-based Jaccard similarity using a combination of minimizers and MinHash (13). MetaMaps adds a mapping quality model and expectation maximization estimation of sample composition. This structure provides faster classification than traditional exact alignment approaches, but retaining the interpretable information of the same. CDKAM (14) is a recent addition to this space that leverages an ‘approximate matching’ strategy for k-mers that enables increased performance on high-error rate sequencing data. This algorithm was also shown to have higher performance than competitors specifically in the middle read lengths (e.g., 1-1.5 kb).

**Text S4:**

Appropriate detection thresholds rely on both (i) pathogen levels associated with an increased risk and (ii) the LOD (limit of detection) or probability of detection of analyte in a defined matrix (15, 16) One method is to treat a similarity match of an amplicon to a reference sequence as a dynamic variable and determining the probability of detection (POD) which is the variance of the LOD. The POD can be evaluated for any method with a sample set of 12 for any concentration level of analyte in a defined matrix (15, 16). This value can be a better predictor of a match between reference and target than a non-parametric system (false positive, false negative rates).

**Text S5**

Users must also consider how to treat ambiguous results based upon an organism’s risk and historical data. Ambiguous results may be deduced from historical samples before, during, and after incidents where a pathogen is clearly detected, but adverse cases cannot be attributed with confidence to the presence of the pathogen. Clearly, such data will not be available in all cases. However, “false alarms” can strongly erode confidence in these monitoring systems (17, 18) so developers have a strong motivation to decrease type I errors using existing samples, data, and information.

**References:**

1. Li B, Saingam P, Ishii S, Yan T. 2019. Multiplex PCR coupled with direct amplicon sequencing for simultaneous detection of numerous waterborne pathogens. Appl Microbiol Biotechnol 103:953–961.

2. Player R, Verratti K, Staab A, Bradburne C, Grady S, Goodwin B, Sozhamannan S. 2020. Comparison of the performance of an amplicon sequencing assay based on Oxford Nanopore technology to real-time PCR assays for detecting bacterial biodefense pathogens. BMC Genomics 21:166.

3. Altschul SF, Gish W, Miller W, Myers EW, Lipman DJ. 1990. Basic local alignment search tool. J Mol Biol 215:403–410.

4. Langmead B, Salzberg SL. 2012. Fast gapped-read alignment with Bowtie 2. Nat Methods 9:357.

5. Li H. 2013. Aligning sequence reads, clone sequences and assembly contigs with BWA-MEM. arXiv:1303.3997. arXiv.

6. Li H. 2018. Minimap2: pairwise alignment for nucleotide sequences. Bioinformatics 34:3094–3100.

7. Kim D, Song L, Breitwieser FP, Salzberg SL. 2016. Centrifuge: rapid and sensitive classification of metagenomic sequences. Genome Res 26:1721–1729.

8. Wood DE, Salzberg SL. 2014. Kraken: ultrafast metagenomic sequence classification using exact alignments. Genome Biol 15:R46.

9. Ounit R, Wanamaker S, Close TJ, Lonardi S. 2015. CLARK: fast and accurate classification of metagenomic and genomic sequences using discriminative k-mers. BMC Genomics 16:1–13.

10. Wood DE, Lu J, Langmead B. 2019. Improved metagenomic analysis with Kraken 2. Genome Biol 20:257.

11. Dilthey AT, Jain C, Koren S, Phillippy AM. 2019. Strain-level metagenomic assignment and compositional estimation for long reads with MetaMaps. 1. Nat Commun 10:3066.

12. Takahashi K, Shimojo A, Matsumoto S, Nakamura M. 2012. MashMap: Application Framework for Map-Based Visualization of Lifelog with Location, p. 1–6. *In* 2012 9th Asia-Pacific Symposium on Information and Telecommunication Technologies (APSITT).

13. Ondov BD, Treangen TJ, Melsted P, Mallonee AB, Bergman NH, Koren S, Phillippy AM. 2016. Mash: fast genome and metagenome distance estimation using MinHash. Genome Biol 17:132.

14. Bui V-K, Wei C. 2020. CDKAM: a taxonomic classification tool using discriminative k-mers and approximate matching strategies. BMC Bioinformatics 21:468.

15. International Organization for Standardization. 2019. Molecular biomarker analysis — Determination of the performance characteristics of qualitative measurement methods and validation of methods. 16393.

16. Wehling P, LaBudde RA, Brunelle SL, Nelson MT. 2011. Probability of Detection (POD) as a Statistical Model for the Validation of Qualitative Methods. J AOAC Int 94:335–347.

17. Afshinnekoo E, Meydan C, Chowdhury S, Jaroudi D, Boyer C, Bernstein N, Maritz JM, Reeves D, Gandara J, Chhangawala S, Ahsanuddin S, Simmons A, Nessel T, Sundaresh B, Pereira E, Jorgensen E, Kolokotronis S-O, Kirchberger N, Garcia I, Gandara D, Dhanraj S, Nawrin T, Saletore Y, Alexander N, Vijay P, Hénaff EM, Zumbo P, Walsh M, O’Mullan GD, Tighe S, Dudley JT, Dunaif A, Ennis S, O’Halloran E, Magalhaes TR, Boone B, Jones AL, Muth TR, Paolantonio KS, Alter E, Schadt EE, Garbarino J, Prill RJ, Carlton JM, Levy S, Mason CE. 2015. Geospatial Resolution of Human and Bacterial Diversity with City-Scale Metagenomics. Cell Syst 1:72–87.

18. McIntyre ABR, Ounit R, Afshinnekoo E, Prill RJ, Hénaff E, Alexander N, Minot SS, Danko D, Foox J, Ahsanuddin S, Tighe S, Hasan NA, Subramanian P, Moffat K, Levy S, Lonardi S, Greenfield N, Colwell RR, Rosen GL, Mason CE. 2017. Comprehensive benchmarking and ensemble approaches for metagenomic classifiers. Genome Biol 18:182.
